# Two matings lead to more copulatory wounding than a single mating in female *Drosophila melanogaster*

**DOI:** 10.1098/rspb.2025.0523

**Published:** 2025-06-18

**Authors:** Bengisu S. Subasi, Anika L. Finsterbusch, Martha Büge, Sophie A. O. Armitage

**Affiliations:** ^1^ Institute of Biology, Freie Universität Berlin, Berlin, Germany; ^2^ Faculty of Life Sciences, University of Vienna, 1190 Vienna, Austria

**Keywords:** copulatory wounding, genital wounding, scar, traumatic mating, traumatic penetration, abdominal wounds

## Abstract

Copulation can result in males inflicting wounds to the female genitalia, so-called traumatic mating. Such wounds are potentially costly as they could be entry points for infections, and they have been associated with a shorter lifespan in insect species. In many species of insects, females mate with more than one male, which leads to the question of whether the number of matings affects the amount of genital damage that females suffer from those matings. Here, we test whether copulation frequency affects the number or size of genital wounds in *Drosophila melanogaster*. Females that mated twice had more genital wounds and a larger total area of wounding, compared with females that mated once. However, females that refused to mate a second time had a similar area of wounding to females that mated twice. We found that wounds to the ventral abdomen also increased with increased mating frequency. Our results show that polyandry can result in increased female copulatory wounding in this species. The extent to which the wounds are costly for females is currently unknown. Investigating genital and abdominal wounds is crucial to a better understanding of the consequences of sexual conflict and the selective pressures shaping mating behaviour.

## 1. Introduction

The evolution of traumatic mating, i.e. the wounding of a mating partner by specialized reproductive structures during copulation [1], is one potential consequence of sexual conflict. Traumatic mating is phylogenetically widespread across animal taxa [1,2]. It is hypothesized to have evolved, e.g. to allow male anchorage to the female during mating or to spur female reproductive investment [1]. In insects, the resulting wounds are repaired by the immune system, which results in melanization at the site of wounding [3]. Melanized patches have therefore been used as indicators of wound repair in the genital tracts of several insect groups, e.g. beetles [4,5], ants [6,7], bedbugs [8] and flies [9,10]. Such copulatory wounds will be costly for the female, e.g. if they provide an opportunity for microbes to enter the body [11–13], if they entail repair costs, or if they result in haemolymph loss [2].

Polyandry is common across animal species [14], and it can benefit females by e.g. increasing reproductive output [15] or by allowing genetic bet-hedging [16]. However, there are also well documented costs, and in insects these can include reduced hatching rate [17] and reduced lifespan ([4,18], but see [19]). Copulatory wounding can be an additional cost of mating in general, e.g. female bean weevils with a higher number of genital scars had shorter lifespans [20]. Although selection under polygamous conditions leads to males that scar females more [20], there is a paucity of studies that have quantified wounding in relation to a known number of matings (but see [8], which quantified the number of wounds to the spermalege, a paragenital organ of female bedbugs). Here, we hypothesized that copulatory wounding is affected by mating frequency, and we predicted that females mating with two males would receive more copulatory wounding compared with females mating with one male.

**Figure 1.**
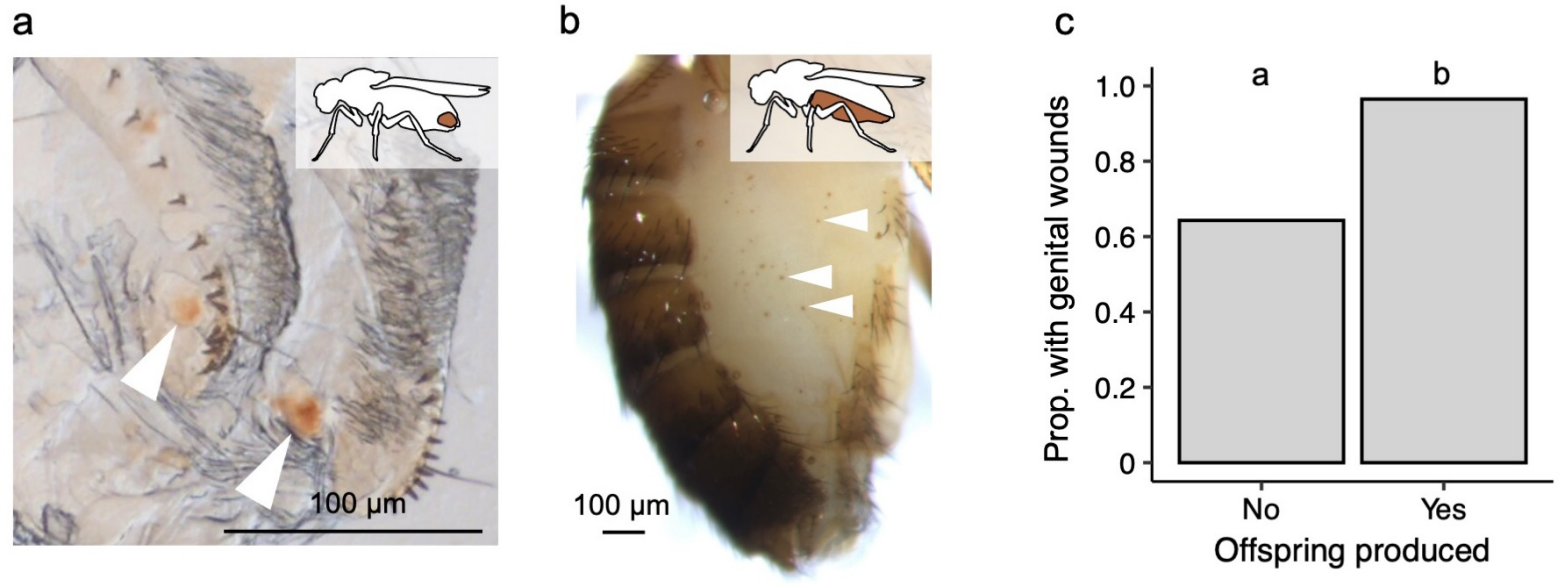
Photos of genital and abdominal wounds, and abdominal wounding prevalence in wild-collected flies. (a) Genital wounding: white arrowheads point to mating-induced wounding (melanized brown patches) on the female vaginal furcal dorsolateral fold; inset image indicates genital region. (b) Abdominal wounding: an example of abdominal wounds on a wild-collected female *D. melanogaster*. White arrowheads point to three of the spots; inset image indicates the abdominal region checked for wounds. (c) The proportion of wild-collected females with wounded genital tracts in relation to whether they produced offspring or not after being brought into the lab. Sample sizes of all females that did not, and that did, produce offspring: *n* = 28 and 57, respectively. The different letters above the treatment groups indicate that the proportions are significantly different from each other.

Female *Drosophila melanogaster* typically mate with more than one male, and they are wounded during copulation. At least 23 species of the *D. melanogaster* species group have evolved traumatic mating [21], including *D. melanogaster* [10,21]. The traumatic mating strategy has since been ascribed to the sub-strategy of traumatic penetration, i.e. where copulatory wounding occurs without the direct injection of male-derived secretions [1]. In *D. melanogaster,* conformational changes of the male phallic parts occur during copulation, which results in genital wounding (figure 1a). The spiny ends of the left and right dorsal branches of the basal processes (more recently termed dorsal postgonites [22]) penetrate the female reproductive tract [21], and they have been hypothesized to play a role in anchoring the male to the female [21]. In *D. melanogaster,* the usually twinned melanized patches indicative of wounding are found on or near the female lateral folds [21], more recently termed the vaginal furcal dorsolateral folds [23]. Such genital damage has been found in wild-collected [24] and lab-reared flies [10]. Furthermore, for more than a century it has been known that female *D. melanogaster* can mate with more than one male under lab conditions [25], and that wild female *D. melanogaster* frequently mate with more than one male [26–29].

Here, we first investigated wounding prevalence in wild-collected *D. melanogaster* females, and its association with offspring production. We predicted that females producing offspring would be more likely to be wounded compared with females with no offspring. We then carried out two lab experiments to test whether copulation frequency affects the number or area of female *D. melanogaster* genital wounds. Harshman & Clark [28] estimated that for 19 wild-inseminated females, the mean number of males that mated with each female was 1.82; we therefore compared singly and doubly mated females. In our first experiment, we predicted that females mated to two males would have more wounds and a larger total area of wounding compared with females mated to one male. There is genetic variation in the amount of harm (courtship and accessory gland proteins in seminal fluid) that male *D. melanogaster* inflict on females, which was measured as the impact on fecundity [30]; therefore we used two *D. melanogaster* populations to test whether there is population-level variation in genital wounding. It was previously observed that wild-collected female *D. melanogaster* can have small melanized spots on their ventral abdomen ([24]; figure 1b), which we hypothesized to be related to mating. To test this, we noted the occurrence of this abdominal wounding on mated and virgin females. Many females in the first experiment did not remate when given the opportunity. Therefore, in our second experiment, we tested whether this self-selecting group of females had a similar degree of genital wounding to singly mated females, or whether they showed intermediate or similar wounding to the doubly mated females. To investigate the relationship between abdominal wounding and mating in more depth, we recorded the location and the number of abdominal wounds of all groups of females.

## 2. Material and methods

### (a) Traumatic mating in wild-collected females

We examined wild-collected females for wounds in their genital tracts, and whether wounding relates to offspring production. Females morphologically identified as *D. melanogaster* and *Drosophila simulans* were collected in early summer, late summer and autumn of 2021, at three sites in Berlin and Brandenburg, Germany (see electronic supplementary material). After collection, the females were placed into individual food vials for 6 days, and we noted whether they produced offspring or not (presence of larvae), as a measure that they had successfully mated. After 6 days, the females were placed individually into microcentrifuge tubes containing 99% ethanol and stored at −20°C for later analysis. We collected 517 females in total, from which 432 produced offspring and 85 did not (see electronic supplementary material, table S1, for sample sizes according to the collection site and season). Of these females, from across the collection seasons and sites, we randomly selected females that did and did not produce offspring (*n* = 58 and *n* = 47, respectively). We examined the genitalia of the male offspring to discriminate between *D. melanogaster* and *D. simulans*. Females that did not produce offspring were identified molecularly (see electronic supplementary material) after they had been dissected to examine copulatory wounds. The methods were the same as described for the below lab experiments, except we recorded whether wounding was present or absent, and not the number or area of wounds. Female genitalia were dissected in October 2022 in a random order and blind with respect to sample collection place and time.

### (b) Experiment 1—The effects of copulation frequency and population on genital wounding and the presence of abdominal wounding

Here, we assessed the effects of copulation frequency (single or double mating) and population origin on the number and area of genital wounds, and on the presence of abdominal wounds. We used females that mated either once or twice and dissected the female genitalia to quantify wounding. We repeated the experiment described below three times between September and October 2022, with each experimental replicate being carried out at two-week intervals. We note that it was previously suggested that *D. melanogaster* copulatory wounds do not show evidence of multiple matings [21], although it was not mentioned how, or whether, wounding was quantified.

#### (i) Fly populations and maintenance

We used two *D. melanogaster* populations. One was established in 2007 from 160 *Wolbachia*-infected fertilized females collected in Azeitão, Portugal [31], gifted to us by Élio Sucena (University of Lisbon), and hereafter referred to as the PT population. The second population was established in 2021 from 402 fertilized females collected from three sites (see electronic supplementary material) in Berlin and Brandenburg, Germany (hereafter the DE population). The populations were maintained in population cages containing at least 5000 flies, with non-overlapping generations of 14 days. We fed the flies with sugar–yeast–agar medium (SYA medium: 970 ml water, 100 g brewer’s yeast, 50 g sugar, 15 g agar, 30 ml 10% Nipagin solution and 3 ml propionic acid [32]), and maintained them at 25°C, on a 12 : 12 h light–dark cycle, at 70% relative humidity. The experimental flies were kept under the same conditions. For two generations prior to the start of each replicate experiment, we reared the flies at a constant larval density of 100 larvae per food vial (95 × 25 mm) containing 7 ml of SYA medium (see electronic supplementary material). Using CO_2_ for anaesthetization, virgins were collected and placed into fresh food vials containing either 10 females or 20 males.

#### (ii) Experimental setup

In total, we had four treatment groups (electronic supplementary material, table S2) (the subscript ‘d’ refers to the number of days post adult eclosion): (i) 0_d5_: control (virgin) females that were not allowed to mate on day 5, (ii) 0_d7_: control (virgin) females that were not allowed to mate on day 7, (iii) 1_d5_: single-mated females that mated once on day 5, and (iv) 2_d5 d7_: double-mated females that mated once on day 5 and again on day 7. Females were randomly assigned to be control females or females given the opportunity to mate. In the afternoon before the first mating, 4-day-old adult virgin males were placed individually into food vials. The following day, starting at 09.00, one person transferred 5-day-old females individually into vials containing a male from the same population as the female, alternating between setting up pairs from the two populations. Females from each population were randomly allocated to the single-mating treatment (1_d5_), with sample sizes per experimental replicate as follows: replicate 1, *n*_DE_ = 123, *n*_PT_ = 114; replicate 2, *n*_DE_ = 103, *n*_PT_ = 103; and replicate 3, *n*_DE_ = 126, *n*_PT_ = 125. At the same time as the mating females, we set up 10 virgin females (0_d5_) per population and experimental replicate (except for seven in the PT population in replicate 2) that were each added to a food vial that did not contain a male.

Immediately after adding a female to a vial, we transferred it to an evenly lit observation shelf. A second person, who was blind to the identity of the population of origin, observed the vial to record the time of transfer, and the time when the mating started and ended. From these data, we calculated the latency to mate (time from transfer to start of mating) and the duration of mating. We gave each pair 3 h to start mating. Males were removed from the vials after copulation to prevent remating. Females randomly allocated to the double-mating group had the following sample sizes: replicate 1, *n*_DE_ = 74, *n*_PT_ = 62; replicate 2, *n*_DE_ = 64, *n*_PT_ = 70; and replicate 3, *n*_DE_ = 84, *n*_PT_ = 66. Approximately 48 h after their first mating, we transferred the double-mating group to food vials containing a virgin male, and the procedure was the same as for the first mating. Once again, we included 10 virgin females per population and replicate (except for seven in the PT population in experimental replicate 2) to be virgin controls (0_d7_) for the second mating. Twenty-four (±1) hours after the end of the first or second copulation, the mated females were anaesthetized using CO_2_ and placed individually into 1.5 ml reaction vials, each containing 100 μl of 99% ethanol. To examine whether the copulation had been successful, we kept the food vials that had contained the females in the 24 h following copulation, and 8 days later, we recorded whether larvae were present.

#### (iii) Examination of genital wounds

One hour (±30 min) after being placed in ethanol, females were removed one by one and transferred to a microscope slide with a drop of *Drosophila* Ringer’s solution (182 mM KCl, 46 mM NaCl, 3 mM CaCl_2_, 10 mM Tris HCl; pH 7.2 [33]; and placed under a stereomicroscope (Leica M205C) at up to 160× magnification. We applied pressure to the female abdomen so that the terminalia were extruded, a second pair of forceps was used to gently pull the terminalia away from the abdomen, and the terminalia were placed onto another microscope slide with 5 μl of Ringer’s solution. A cover slip was placed on top to flatten out the terminalia to examine the vaginal furcal dorsolateral folds. The person observing melanized spots and dissecting the flies was blind to the identity of the flies and their treatment, and they were processed in a randomized order. Sample sizes of females successfully dissected and photographed per experimental replicate and population were as follows: for 0_d5_: replicate one, *n*_DE_ = 6, *n*_PT_ = 6, replicate two, *n*_DE_ = 7, *n*_PT_ = 4, replicate three, *n*_DE_ = 4, *n*_PT_ = 7; for 0_d7_: replicate one, *n*_DE_ = 8, *n*_PT_ = 10, replicate two, *n*_DE_ = 9, *n*_PT_ = 6, replicate three, *n*_DE_ = 7, *n*_PT_ = 8; for 1_d5_: replicate one, *n*_DE_ = 16, *n*_PT_ = 14, replicate two, *n*_DE_ = 11, *n*_PT_ = 13, replicate three, *n*_DE_ = 7, *n*_PT_ = 6; and for 2_d5 d7_: replicate one, *n*_DE_ = 5, *n*_PT_ = 13, replicate two, *n*_DE_ = 11, *n*_PT_ = 7, replicate three, *n*_DE_ = 8, *n*_PT_ = 3. We visualized the dissected genitalia using a light microscope (Axiophot, Zeiss, Germany) and one photograph was taken of each tract using a Jenoptik ProgRes CF digital camera, at a total magnification of 250×. The photographs were analysed using ImageJ [34] version 1.53 a. Wounds appear as brown spots on the genital tract ([10] and see figure 1a). We recorded the number of wounds and their total area on one side of the vaginal furcal dorsolateral folds, as in most photographs only one side was visible. In preliminary tests, we did not find significant differences between the average of measuring two sides compared with the measurement from one side only (see electronic supplementary material). We traced the outline of each wound and the total area per female was recorded in μm^2^. The repeatability of measuring the area of the wounds with the above analysis methods was high (see electronic supplementary material). A preliminary experiment showed that the area of the wound did not change significantly if females were sacrificed 1, 3 or 6 days after mating (electronic supplementary material, figure S1).

#### (iv) Examination of abdominal wounds

A previous study showed that wild-collected females exhibit more melanized spots on their ventral abdomen compared with males, and that similar wounds are absent on the female dorsal abdomen and in males [24]. Based on these findings, we focused exclusively on the ventral abdomen to test whether there is an association between melanized spots and mating. After mating and prior to genital dissection, females were placed in a drop of *Drosophila* Ringer’s solution and examined under a stereomicroscope (Leica M205C) at up to 160× magnification. We carefully inspected the ventral abdomen of mated and virgin females and recorded the presence/absence of melanized spots in experimental replicates 2 and 3.

### (c) Experiment 2—The effects of single matings, double matings, and not remating, on genital and abdominal wounding

The first set of experiments resulted in a group of self-selecting females that did not mate a second time, and in this second experiment we asked whether this group were wounded to a similar degree to the singly or doubly mated females, or whether they had an intermediate amount of wounding. Our second aim in this experiment was to examine abdominal wounding in relation to mating, by recording the location and the number of abdominal wounds in all female treatment groups. We used only the PT *D. melanogaster* population, and the maintenance of the flies and experimental procedures followed the same methods as described above. We repeated the experiment described below three times in July 2024, with one experimental replicate being carried out per week.

In addition to the previously established treatment groups in experiment 1, two additional groups were included (electronic supplementary material, table S3): (v) 1_d7_, females mated once on day 7 to control for possible differences associated with female aging; and (vi) 1_d5, no d7_, females mated on day 5 but that did not remate on day 7 despite being exposed to a male, i.e. the self-selecting group of females that did not remate. Ten females per replicate were randomly allocated to the virgin control group (0_d7_). In each experimental replicate, females were randomly allocated to single-mating treatments (1_d5_ and 1_d7_), with sample sizes as follows: replicate one, *n* = 176; replicate two, *n* = 267; and replicate three, *n* = 379. For the double mating treatment (2_d5 d7_), the numbers of females allocated per experimental replicate were: replicate one, *n* = 112; replicate two, *n* = 121; and replicate three, *n* = 187. Of the females that refused to mate a second time, 15 were randomly allocated to the group that mated on day 5 but did not remate on day 7 (1_d5, no d7_). Samples sizes of females successfully dissected and photographed per experimental replicate were as follows: for the single mating treatments: 1_d5_: replicate one, *n* = 6, replicate two, *n* = 19, replicate three, *n* = 14; 1_d7_: replicate one, *n* = 14, replicate two, *n* = 13, replicate three, *n* = 21; 1_d5, no d7_: replicate one, *n* = 16, replicate two, *n* = 15, replicate three, *n* = 14; and for 2_d5 d7_: replicate one, *n* = 10, replicate two, *n* = 6, replicate three, *n* = 13.

To determine whether melanized spots on the ventral abdomen were caused by mating, flies in some treatment groups were examined both before and after mating (see electronic supplementary material, table S3). Unlike experiment 1, females were examined for abdominal wounds prior to the first mating. On the day before the first mating, 4-day-old adult virgin females were briefly anaesthetized with CO_₂_ and assessed for abdominal wounds. To do this, females were placed on a small piece of Parafilm, and a drop of water was applied to their abdomens to enhance the visibility of melanized spots. Preliminary tests confirmed that the water did not influence melanized spot formation. Each female was examined under a stereomicroscope (Leica M205C) at up to 160× magnification. We noted on which of tergite numbers 1–7 the melanized spots were located, and how many spots each female had. After this the females were placed individually into numbered vials containing fresh food.

The following day, mating was carried out as described in experiment 1. Twenty-four hours after the first mating, 0_d5_ and 1_d5_ females were again examined for abdominal wounding. After examining the abdomen, mated females were dissected to quantify copulatory wounds as described above. Before the mating on day 7, females from the 0_d7_ and 1_d7_ groups were examined for abdominal wounds. After the second mating opportunity, females from the 1_d5, no d7_ and 2_d5 d7_ were examined for melanized spots, and dissections were performed to assess copulatory wounds.

### (d) Statistical analyses

To perform the analyses, R [35] version 4.2.2 and RStudio [36] version 2024.09.0+365 were used. Experimental replicate was included as a random factor in each model, unless stated otherwise. We fitted generalized linear mixed models (glmms) using the glmmTMB package [37] and lme4 [38]. For residual diagnostics of the statistical models, the DHARMa package [39] was used. The car package [40] was employed for type II ANOVAs. *Post hoc* multiple comparisons were performed with the fifer [41] or emmeans [42] packages, using the Bonferroni adjustment for multiple comparisons. Data visualization was carried out using ggplot2 [43]. We also used the following packages for visualization and data summaries: dplyr [44], PairedData [45], plyr [46], purrr [47], plotrix [48], qqplotr [49] and tidyr [50]. Details of all models are given in the electronic supplementary material, and the datasets and R script are available on Dryad [51].

## 3. Results

### (a) Wild-collected females show genital wounding

Eighty one per cent (*n* = 85) of the randomly selected wild-collected females were *D. melanogaster*. Of the *D. melanogaster* females, 86% had genital wounds. Wild-collected females that produced offspring in the lab were more likely to be wounded than females that did not produce offspring (*χ*^2^ = 11.03, d.f. = 1, *p* = 0.0009; figure 1c). Indeed 97% of females that produced offspring had visible wounds, but we note that a substantial proportion of females that did not produce offspring in the lab also had visible wounds (figure 1c).

### (b) Lab experiments

#### (i) Proportions of females mating at the first and second mating attempts

In the first experiment, 81.3 ± 2.95% (s.e.) of females copulated at the first mating opportunity, and around 13 ± 2.43% (s.e.) of females mated a second time. There was no effect of population on the frequency of females that mated at the first (*χ*^2^ = 3.70, d.f. = 1, *p* = 0.055) or second mating (*χ*^2^ = 0.20, d.f. = 1, *p* = 0.658). In the second experiment, 62.3 ± 5.19% (s.e.) of females copulated at the first mating opportunity, whereas only 3.6 ± 1.27% (s.e.) of females mated a second time.

#### (ii) Latency to mate was longer for the second mating

Latency to mate was significantly longer for the second mating compared with the first mating in both experiments 1 and 2 (electronic supplementary material, table S4 and figure S2a,b). In experiment 1, the DE population started mating more quickly than the PT population (electronic supplementary material, table S4). In contrast to latency, in experiment 1, copulation duration was marginally longer for the first mating compared with the second mating, but it did not differ in experiment 2 (electronic supplementary material, table S4 and figure S2c,d).

#### (iii) Genital wounding is affected by mating frequency

As expected, none of the virgin females (*n*_exp1_ = 82) but all of the mated females (*n*_exp1_ = 114) had visible genital wounds. In the subset of females that mated once, for both experiments, there was no effect of latency to mate or copulation duration on the number of wounds, or the area of wounding (electronic supplementary material, table S5).

**Figure 2.**
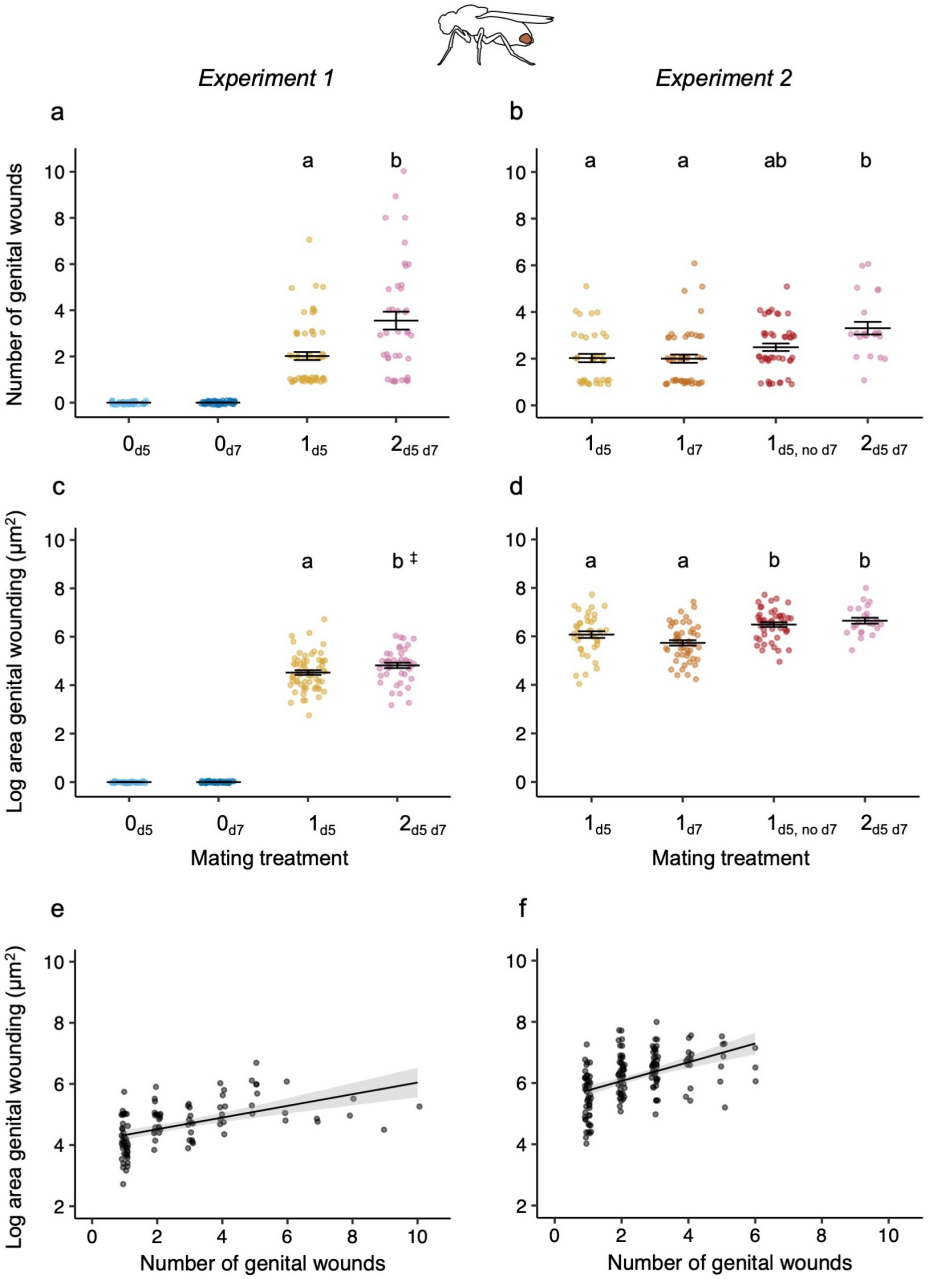
The effect of the number of matings on genital wound number and area. The effect of the number of matings on the number of wounds observed for (a) experiment 1 and (b) experiment 2, and on the natural-log-transformed area of wounding for (c) experiment 1 and (d) experiment 2. The relationship between the number and area of genital wounds for (e) experiment 1 and (f) experiment 2. Experiment 1 sample sizes for the number of wounds: 0_d5_ = 34, 0_d7_ = 48, 1_d5_ = 61, 2_d5 d7_ = 40, and for area: 0_d5_ = 34, 0_d7_ = 48, 1_d5_ = 61, 2_d5 d7_ = 41. Experiment 2 sample sizes for both variables: 1_d5_ = 39, 1_d7_ = 49, 1_d5, no d7_ = 45 and 2_d5 d7_ = 23. For (a,c), the virgin treatment groups are included for visualization but were not included in statistical tests because they showed no wounding. To visualize wound area, all raw values had 1 added to them before log transformation. When considering the mated treatments, only females that mated for a minimum of 10 min and that produced larvae after the first mating are included. Each data point is from one female and to enable visualization of all data points they are jittered for the number and area of wounds and the treatment groups. Means and s.e. are shown. When letters above the treatment groups are not the same, the treatment groups are significantly different from each other. ^‡^When the area of wounding was analysed including females that did not produce larvae, *p* = 0.058; see table 1.

As predicted, across both experiments females that mated twice received more wounds compared with singly mated females (table 1; figure 2a,b). Furthermore, the females that did not remate (1_d5, no d7_) had a similar number of wounds to both single- and double-mated females (electronic supplementary material, table S6; figure 2b). Wound area showed a similar effect of mating frequency to wound number, i.e. the wound area was larger when females mated twice compared with once (electronic supplementary material, table S6; figure 2c,d). However, in contrast to wound number, wound area of the females that did not remate (1_d5, no d7_) was significantly larger than that of singly mated females, but similar to that of the females that mated twice (electronic supplementary material, table S6; figure 2d). In experiment 1, neither wound number nor wound area was affected by the fly population or an interaction between mating treatment and fly population (table 1). Across both experiments, there was a positive relationship between the total number of wounds and the area of wounding (table 1; figure 2e,f).

**Table 1.** The effects of mating frequency and population on genital wound number and area. Each response variable was first tested using only females that produced larvae after the first mating, and then using all females, i.e. also females that did not produce larvae after the first mating. Sample sizes are given in the table. Bold type indicates statistically significant effects

experiment	response variable	tested effect	d.f.	*χ* ^2^	*p*
1	wound number (*n =* 101; only females producing larvae)	mating treatment	1	20.20	**<0.0001**
		population	1	0.73	0.39
		mating treatment × population	1	1.54	0.21
	wound number (*n =* 110; females that did and did not produce larvae)	mating treatment	1	18.05	**<0.0001**
		population	1	0.94	0.33
		mating treatment × population	1	2.04	0.15
	log wound area (*n =* 102; only females producing larvae)	mating treatment	1	4.22	**0.040**
		population	1	0.60	0.44
		mating treatment × population	1	2.28	0.13
	log wound area (*n =* 112; females that did and did not produce larvae)	mating treatment	1	3.59	0.058
		population	1	0.46	0.50
		mating treatment × population	1	2.05	0.15
	log wound area (*n =* 101; only females producing larvae)	wound number	1	22.18	**<0.0001**
		(wound number)^2^	1	9.91	**0.0016**
		mating treatment	1	0.00	1.00
		wound number × mating treatment	1	0.23	0.63
		(wound number)^2^ ×mating treatment	1	0.05	0.81
	log wound area (*n =* 110; females that did and did not produce larvae)	wound number	1	20.90	**<0.0001**
		(wound number)^2^	1	8.91	**0.0028**
		mating treatment	1	0.07	0.79
		wound number × mating treatment	1	0.23	0.63
		(wound number)^2^ × mating treatment	1	0.04	0.84
2	wound number (*n* = 132)	mating treatment	3	20.05	**0.00017**
	log wound area (*n =* 132)	mating treatment	3	39.33	**<0.0001**
	log wound area (*n =* 132)	wound number	1	18.01	**<0.0001**
		(wound number)^2^	1	7.64	**0.0057**
		mating treatment	3	21.92	**<0.0001**
		wound number × mating treatment	3	3.00	0.39
		(wound number)^2^ × mating treatment	3	3.48	0.32

#### (iv) Abdominal wounding is affected by mating frequency

**Figure 3.**
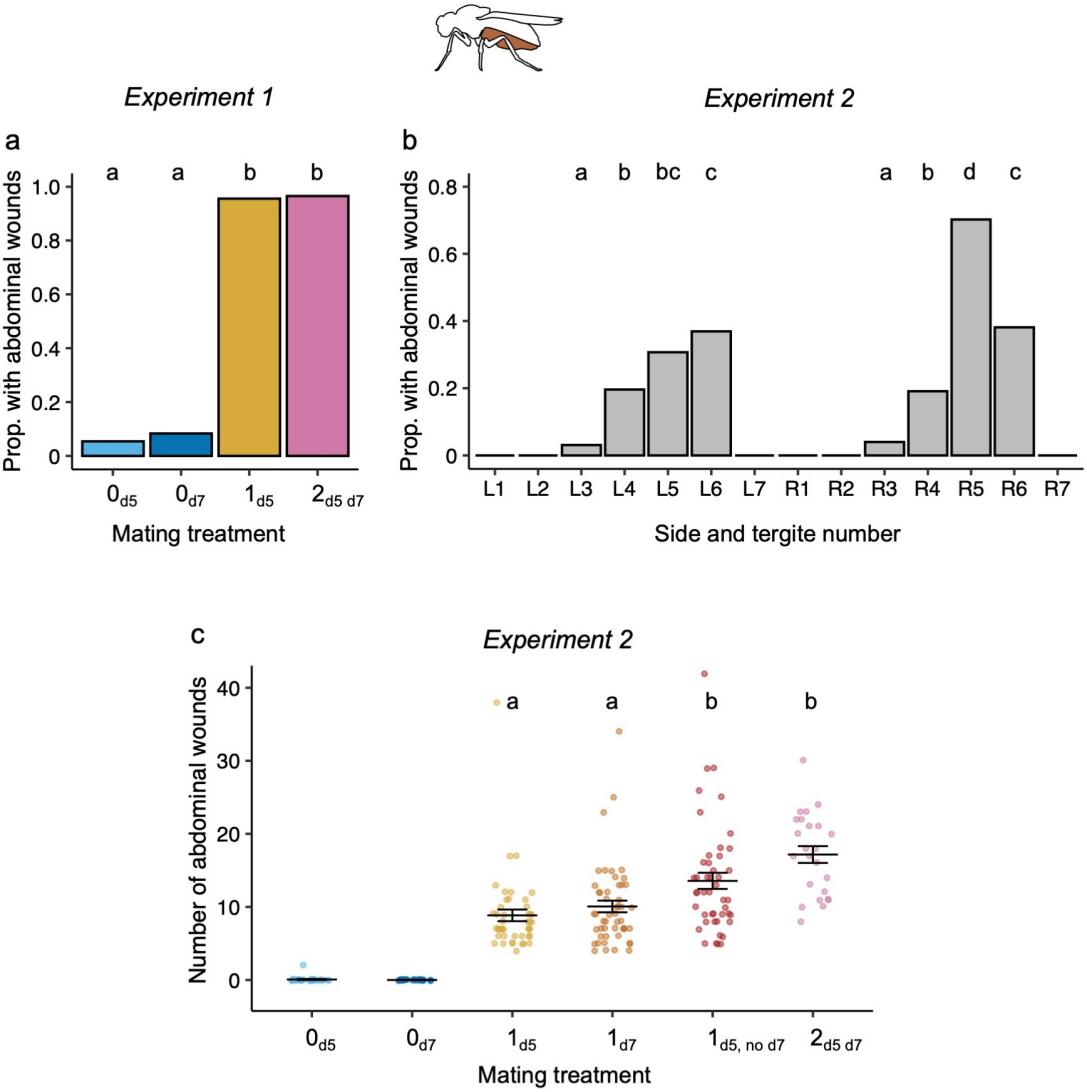
The location of abdominal wounds and the effect of the number of matings on abdominal wound frequency**.** Experiment 1: (a) The proportion of virgins (0) and females mating once (1) or twice (2) that had melanized spots visible on the exterior of their ventral abdomens. Sample sizes of all females in each treatment group: 0_d5_ = 37, 0_d7_ = 36,1 _d5_ = 68, 2_d5 d7_ = 29. Here and elsewhere, when letters above the treatment groups are not the same, the treatment groups are significantly different from each other. Experiment 2: (b) The proportion of females that had abdominal wounds on their tergites [1–7], where 1 refers to the tergite closest to the thorax), and whether the spots were on the left (L) or the right (R) side of the tergites. The total sample size is *n* = 235. Note that treatment groups with proportions of 0 (L1, L2, L7, R1, R2 and R7) were not included in *post hoc* multiple testing. (c) The number of ventral abdominal spots found on each female for each of the mating treatments. Sample sizes are: 0_d5_ = 30, 0_d7_ = 30, 1_d5 =_ 45, 1_d7_ = 50, 1_d5, no d7_ = 46 and 2_d5 d7_ = 24. The two virgin groups were not included in the statistical test; hence no letter is given above the data. Each data point is from one female and data points are jittered for the number of abdominal wounds and the treatment groups. Means and s.e. are shown.

In experiment 1, the presence of abdominal wounds on the ventral abdomen was almost completely related to mating treatment: fewer than 1% of virgins had visible abdominal wounds whereas more than 95% of mated females had visible abdominal wounds (figure 3a; electronic supplementary material, table S7). *Post hoc* comparisons showed that females that mated once or twice were more likely to have abdominal wounds than virgin females (electronic supplementary material, table S8).

In experiment 2, we investigated the location and number of abdominal wounds on the ventral abdomen. The abdominal tergites varied significantly in the number of wounds they received following mating (*χ*^2^ = 269.13, d.f. = 7, *p* < 0.0001). The wounds were found exclusively on tergites 3–6, with 70% of females showing wounds on the fifth tergite on the right-hand side of the abdomen (figure 3b). Consistent with experiment 1, abdominal wounds were strongly associated with mating treatment. Only one virgin female had two abdominal wounds before mating, and this number remained unchanged after mating (figure 3c). No singly mated females had wounds before mating and all females had at least four wounds on their abdomen after mating (figure 3c). Overall, there was a significant effect of mating frequency on the number of wounds (electronic supplementary material, table S7). Similar to the area of genital wounds, females that mated twice had more abdominal wounds compared with those mated once (figure 3c; electronic supplementary material, table S8), and the number of wounds on the females that did not remate (1_d5, no d7_) was significantly higher than on singly mated females, and did not differ from the females that mated twice (figure 3c; electronic supplementary material, table S8).

#### (v) No relationship between the number of abdominal wounds and genital wounding

There was no relationship between the number of abdominal wounds and either the number (electronic supplementary material, figure S3a and table S9) or the area of genital wounds (electronic supplementary material, figure S3b and table S9).

## 4. Discussion

Here, we show that almost all wild-collected females that produced offspring had visible genital wounding. In our lab experiments, as predicted, two successful matings resulted in more wounds and a larger total area of wounding to the female genitalia, compared with when females were only allowed to mate once. We furthermore identified ventral abdominal wounding as being almost exclusively found in mated females. Abdominal wounds showed a similar pattern to genital wound area: females that mated twice had more abdominal wounds than those that mated once. Our findings suggest that in *D. melanogaster*, copulatory wounding is correlated with polyandry, which may be a potential unexplored cost of multiple mating.

A previous study of wild-collected females showed that 98.4% had sperm in their reproductive tracts, i.e. had mated [52]. Consistent with this number, here we show that of the females that produced larvae, 97% were wounded. However, we also found that many wild-collected females had genital wounds but did not produce offspring (64%). Given that the results from the lab show that virgins do not have wounds (this study and [10]), it seems unlikely that the 64% of females were unmated; instead it seems more probable that the conditions in the lab were not conducive for producing offspring, or that females had exhausted their sperm supplies.

Traumatic mating is one expression of sexual conflict, e.g. a single mating with a male seed beetle, *Callosobruchus maculatus*, evolved under conditions with increased sexual conflict (polygamy), resulted in more female copulatory damage compared with mating with a male evolved under monogamous conditions [20]. We here show that females mating twice had more wounds, and a greater area of wounds compared with females that mated once. We are only aware of two other studies that have tested whether female genital wounding in insects is affected by mating frequency when mating number is controlled: Blanckenhorn *et al.* [9] found that the proportion of female dung flies, *Sepsis cynipsea*, with genital wounds was higher after two copulations (approx. 30%) compared with after one copulation (10%), and Bartonička *et al.* [8] found a positive relationship between the number of matings and the number of scars to the paragenital organ of female common bedbugs, *Cimex lectularius*. The increase in proportion or number of wounds with increasing mating number is consistent with our findings when comparing treatments 1_d5_ and 1_d7_ with the 2_d5 d7_ treatment. However, in *D. melanogaster* every mating results in at least one scar ([10]; present study), whereas not every dung fly copulation resulted in wounding [9], and on average 3.5 matings led to one bedbug scar [8]. As might be predicted, and similar to Gay *et al*. [20], we found a positive correlation between wound area and the number of genital wounds. Questions arising from these results are whether the wounding we observed imposes physiological or life history costs on females, and if so, whether females incur additional costs when they mate twice compared with mating just once. In principle, physiological costs could come from wound repair, which involves the use of the enzyme phenoloxidase (PO) [53–55]. Activation of PO and the subsequent production of melanin results in cytotoxic side products such as reactive oxygen species that can help to kill microorganisms [56,57] but can also harm the host [58], and interestingly in *C. maculatus* enforced monogamy results in the evolution of lower PO activity [59,60]. Additional costs could arise from the fact that copulatory wounds are an entry point for microbes [11–13], and they could result in haemolymph loss [2], both of which could be deleterious for the host. Life history costs could come in the form of, e.g., reduced offspring production or survival, and mating rate has frequently been shown to affect both of these traits (reviewed in [2] ). To disentangle the costs of mating that are specifically associated with copulatory wounding, it would be necessary to consider factors such as male-derived sex peptides and other seminal fluid proteins transferred during mating, which are known to affect female fitness [61,62].

The self-selecting group of females that refused to remate (1_d5, no d7_) showed an intermediate number of wounds between the single- and double-mated females, and a similar area of wounding to the double-mated females and more than the singly mated females. We reasoned that the genital wounding in this group resulted from the first copulation, as these females did not copulate successfully a second time. One hypothesis to arise from these findings is that the increased area of genital wounding in this self-selecting group of females compared with singly mated females is linked to the refusal to remate, for example if larger wounds are more costly to repair and if this affects the remating rate. Alternatively, it is possible that females that are generally more reluctant to mate (whether during the first or second mating) receive more injuries in the first mating owing to increased resistance or prolonged mating struggles. If increased wounding does indeed reduce the propensity of females to remate, a potentially hidden cost of increased genital wounding could be that it limits the benefits of polyandry, such as enhanced offspring genetic diversity and viability [63,64]. To uncover whether damage sustained at mating affects remating behaviour, one would need to consider male-derived peptides that are transferred to females at mating, which can also influence remating behaviour [65–67].

It was previously documented that wild-collected females have more wounds on the ventral abdomen compared with males [24]. Here we show that the wounds were predominantly located between the third and sixth segments on the ventral abdomen, i.e. on the terminal half of the abdomen, under which the female reproductive organs lie. We found that these abdominal wounds are associated with mating: fewer than 1% of virgins, but almost all (>95 %) mated females had visible spots. In line with our findings for genital wounding, females mating twice had more abdominal wounds compared with females that mated once. Furthermore, females that mated once but refused a second mating showed a similar number of abdominal wounds to those that mated twice. However, despite the similar responses of genital and abdominal wounding to mating frequency, we did not detect a positive relationship between the number or area of genital wounding and the number of abdominal wounds, which might suggest that these two areas of wounding are somewhat independent from each other.

Unlike the genital wounds, the abdominal wounds are visible on the exterior of the ventral abdomen. It was previously hypothesized that the ventral abdominal wounds could be due to external damage caused by males during copulation [24]. In *D. melanogaster*, males exhibit a series of courtship behaviours including tapping, following, wing vibrations, licking, abdominal bending, lunging, and grasping of the female [68,69]. During courtship it has also been reported that the tarsi of the male's forelegs are extended under the abdomen of the female and vibrate against it [70], or that the forelegs are used to grasp the abdomen [71]. It is unknown whether any of these behaviours would be able to cause the abdominal wounds that we observed. A non-mutually exclusive alternative explanation is that the abdominal wounds could be due to an internal physiological response to copulation itself [24], which may originate from autoimmune damage elicited by mating or substances/pathogens (e.g. disseminated melanization after pathogen injection, see [72]) transferred at mating. Future studies will be needed to uncover the exact mechanisms.

We observed that the right-hand side of the fifth tergite showed almost double the number of abdominal wounds compared with either side of any of the other tergites. Laterality in mating behaviour or genital asymmetry are both widespread phenomena among insects, including Drosophilidae [73]. For instance, in *Drosophila pachea*, males consistently mate from the right-hand side [74,75]. *Drosophila melanogaster* males also demonstrate mating laterality via unilateral wing vibrations during courtship [76]. However, unlike *D. pachea*, where the males consistently have a right-sided position, in *D. melanogaster* each male can extend either the left or the right wing during courtship, i.e. both wings can be used, but not simultaneously. If there are side biases in mating posture or certain movements, it might explain the right-side dominance in abdominal wounding that we observed in our study. However, further experiments are needed to investigate the generality and reasons for this pattern.

Our experiment also allowed us to examine the proportion of remating females. Previous lab studies have shown that female remating rates can vary considerably according to exposure time to males [77], remating interval [19,78] and genotype [79]. Chow *et al.* [79] found remating rates between *ca* 10 and 100% for the DGRP fly lines that they tested, and the remating rates in the present study are at the bottom end of that distribution; however, there are differences in the experimental set-up used by that study and our own. One potential explanation for the lower remating rates in our second experiment compared with the first experiment could be the fact that the females were anaesthetized with CO_2_ more frequently in the second experiment because of the checks for abdominal wounds, and this form of anaesthetization can increase copulation latency [80]. Consistent with Pavković-Lučić & Kekić [81], we found that latency to mate was longer for the second mating compared with the first mating. Copulation duration was longer for the first mating in experiment 1, which is consistent with some studies [81,82] but not with other studies [83–85].

In conclusion, our findings provide a proof of concept that in *D. melanogaster*, increased mating rate increases female copulatory wounding. Interestingly, females that refused to remate incurred similar damage to twice-mated females, highlighting the importance of investigating this self-selecting group of females. The implications of the wounds, e.g. whether they have measurable costs, and whether or not the higher amount of genital wounding in non-remating females is causally related to the lack of remating, remain to be explored. In *D. melanogaster,* systemic infections can result when females mate with males carrying pathogenic bacteria on their genitalia [86]. The mechanism for the latter is untested, but one possibility is that bacteria pass through copulatory wounds into the haemocoel, as was demonstrated for microbeads in *Drosophila santomea* and *Drosophila yakuba* (albeit at low rates [87]). If this is the case, there is the potential for more numerous or larger total wounds after multiple mating to increase infection risk.

## Data Availability

The datasets generated in this study and the R script containing statistical analyses are available on Dryad [51]. Supplementary material is available online [88].
